# The Dynamic Association Between Physical Activity and Psychological Symptoms in Young People With Major Depressive Disorder: An Active and Passive Sensing Longitudinal Cohort Study

**DOI:** 10.1111/eip.70018

**Published:** 2025-02-23

**Authors:** Rosalind Baynham, Andres Camargo, Simon D'Alfonso, Tianyi Zhang, Zamantha Munoz, Pemma Davies, Mario Alvarez‐Jimenez, Niels van Berkel, Vassilis Kostakos, Lianne Schmaal, Scott D. Tagliaferri

**Affiliations:** ^1^ School of Sport, Exercise and Rehabilitation Sciences University of Birmingham Birmingham UK; ^2^ Centre for Youth Mental Health The University of Melbourne Parkville Australia; ^3^ Orygen Parkville Australia; ^4^ School of Computing and Information Systems The University of Melbourne Parkville Australia; ^5^ Aalborg University Copenhagen Denmark

**Keywords:** actigraphy, affect, depressive symptomatology, ecological momentary assessment, physical activity

## Abstract

**Purpose:**

Physical activity could be associated with psychological symptoms in young people with major depressive disorder (MDD). Using actigraphy and ecological momentary assessment (EMA), we investigated the associations between physical activity and stress, anxiety and positive and negative affect in young people with MDD.

**Methods:**

Actigraphy and EMA were collected daily in 40 young participants (aged 16–25 years) with MDD over 8 weeks. Multi‐level linear mixed models were used to examine within‐ and between‐person daily associations between physical activity and symptoms of stress, depression and positive and negative affect.

**Results:**

Participants with at least 14 days of complete data were included in the analysis (*n* participants = 22; total days = 598). Typical (defined as average across the assessment period) vigorous physical activity was significantly associated with lower daily stress (*β*[95% CI] = −0.152 [−0.298, −0.007], *p* = 0.041) and higher daily positive affect (0.526 [0.061, 0.992], *p* = 0.028). Variability in daily light (0.004 [0.001, 0.006], *p* = 0.010) and moderate physical activity (0.004 [0.001, 0.007], *p* = 0.009) were positively associated with daily stress. Variability in daily light (0.003 [0.001, 0.006], *p* = 0.018) and moderate physical activity (0.004 [0.001, 0.007], *p* = 0.011) were positively associated with daily anxiety.

**Conclusions:**

Various intensities of daily physical activities were associated with symptoms of stress, anxiety and positive affect in young people with MDD. Future research on larger samples should validate the causal and daily associations between physical activity and psychological symptoms to promote evidence‐based behavioural strategies to improve psychological symptoms in young people with MDD.

## 1 Introduction

Depression is a major contributor to the global burden of disease, affecting approximately 400 million people worldwide (World Health Organisation 2023). Depression is a mood disorder, characterised by sadness and loss of interest and energy, which affects thoughts, feelings and behaviours, and can impair physical and psychological health (American Psychiatric Association 2013). For example, depression negatively affects physical health, shown by an increased risk of cardiovascular diseases, mortality, stroke, cancer and Alzheimer's disease (Ownby et al. 2006; Rajan et al. 2020; Ramasubbu and Patten 2003). Furthermore, depression has psychological consequences, as evidenced by its frequent comorbidity with anxiety (Kalin 2020), symptoms of reduced positive and increased negative affect (American Psychiatric Association 2013) and its bidirectional relationship with stress (Hammen et al. 2009; Kay and Tasman 2006). There is increasing attention on depression in adolescents (Zhou et al. 2021), given it is well established that the burden of the condition peaks during adolescence and young adulthood (Kessler et al. 2007). Therefore, research evaluating factors which are associated with physical and psychological health in young people with depression is essential.

Physical activity is promoted for the improvement of physical (Anderson and Durstine 2019) and psychological health (Gianfredi et al. 2020; Wipfli, Rethorst, and Landers 2008), making it an optimal strategy for those with depression. The association between physical activity and symptoms of depression appears to be bidirectional (Azevedo Da Silva et al. 2012). For example, people with depression have lower physical activity levels compared to depression‐free individuals (Minaeva et al. 2020), and physical inactivity increases the risk of depression (Schuch et al. 2016). The effect of physical activity on depression appears convincing, with two meta‐analyses presenting an inverse association between depression and physical activity (Krogh et al. 2017; Schuch et al. 2017), whereby engaging in physical activity can reduce symptoms of depression. However, few studies use objective measures of physical activity (Gianfredi et al. 2020), and most are cross‐sectional (McKercher et al. 2009), have limited durations of data collection (Gianfredi et al. 2020), or do not consider factors such as stress, anxiety and positive and negative affect (Gianfredi et al. 2020), which may influence this relationship (Remes, Mendes, and Templeton 2021). Therefore, overcoming these limitations could provide useful insights into the relationship between physical activity and depression.

One methodology that could be used to examine acute associations and the longitudinal relationship between objective physical activity (measured via passive actigraphy sensing) and psychological symptoms in people with depression is ecological momentary assessment (EMA). EMA is an active sensing method which uses repeated sampling of participant behaviour, feelings and experiences in real‐time (e.g. day‐to‐day), in participants' free‐living environment (Shiffman, Stone, and Hufford 2008). Capturing data in this way enables the consideration of both between‐day (deviation from average group level) and within‐day (deviation from individual's own average) associations across the same period (Dunton 2017). Both physical activity and psychological symptoms of depression can fluctuate day‐to‐day (Schoevers et al. 2021; Shang et al. 2018). Therefore, by using EMA and passive sensing (actigraphy), we can examine the association between fluctuations in physical activity and psychological symptoms. Furthermore, given that adolescence and young age is a critical age for healthy behaviour change (Villafaina et al. 2021), investigating fluctuations in physical activity and psychological symptoms in young people is essential. The feasibility of EMA research in adolescence (Russell and Gajos 2020) and mood disorders (Wenze and Miller 2010) is promising, yet most studies investigating young people include non‐clinical samples (Wen et al. 2017), or do not assess physical activity (Russell and Gajos 2020), making the current study a novel exploration.

Therefore, using actigraphy and EMA, the present study had four aims to investigate the daily associations between physical activity and the symptoms of (a) stress, (b) anxiety, (c) positive affect and (d) negative affect, in a clinically depressed young population. It is hypothesised that physical activity will be associated with reduced symptoms of stress, anxiety, and negative affect, and increased symptoms of positive affect.

## 2 Methods

### 2.1 Study Design

The present longitudinal cohort study was approved by the University of Melbourne Human Research Ethics Committee (ID: 1955691.4). This study was carried out in accordance with the principles contained in the Declaration of Helsinki and the Australian National Health and Medical Research Council National Statement on Ethical Conduct in Human Research, and the NHMRC Australian Code for the Conduct of Research. The data were partly collected during the COVID‐19 pandemic and reported based on Strengthening the Reporting of Observational Studies in Epidemiology guidelines (Elm et al. 2007), which are located in Table S1.

### 2.2 Participants

Forty young people (aged 16–25 years) attending five Victoria *headspace* centres (Melbourne, Australia), with a diagnosis of major depressive disorder (MDD); determined by the treating clinician and the Mini‐International Neuropsychiatric Interview (MINI) interview (Sheehan et al. 1998), were recruited. Exclusion criteria were (i) actively manic or psychotic (based on MINI diagnostic interview), (ii) not able to converse in, or read English, (iii) lack of familiarity with smartphones to complete EMA surveys, (iv) currently at acute risk of suicide (assessed by the treating clinician). We aimed to recruit 40 young people due to the overall resources available to the wider study. For this analysis, we included participants if they had a minimum of 14 days with both EMA and actigraphy data to appropriately model within‐ and between‐person effects over the study period. Participants were reimbursed $100 AUD upon completion of the study, as well as an additional $1.5 for completion of each EMA survey (maximum $126 if all surveys were completed).

### 2.3 Procedures

**FIGURE 1:**
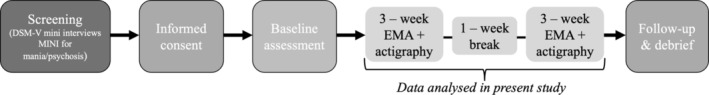
Study flow.

Data collection took place between September 2020 and May 2022. Informed consent was obtained from all participants before enrolment in the study. Participants aged 16–18 years old were also required to have consent from their nominated parent/guardian. Initial screening was undertaken to assess eligibility. If eligible, participants were briefed about the study. For consenting participants, a baseline assessment for demographic measures and baseline depression (as well as other psychological questionnaires, data not reported) was undertaken. Upon completion of the baseline assessment, the AWARE‐Light smartphone sensing app was installed on each participant's (Android) smartphone (Berkel et al. 2023). The AWARE‐Light app was used to collect EMA data, as well as other passive sensing data not reported within this study (e.g. geolocation, screen unlocks, application usage, incoming/outgoing calls). Following the app installation, participants were provided an actigraphy device and instructed not to remove it, to monitor their physical activity and sleep (sleep data not reported in these analyses). Actigraphy and EMA data were collected continuously for 3 weeks, followed by a week break (where the actigraphy device was replaced with a fully charged one), and a second three‐week block of data collection (Figure 1). Days with completed actigraphy and EMA data were included across the 6 weeks, allowing for a more complete data set. Following data collection, participants were debriefed, the AWARE‐Light app was uninstalled from their mobiles and the actigraphy devices were returned.

### 2.4 Variables and Confounders

#### 2.4.1 Demographic Information

Demographic information included measures of age, self‐identified sex and self‐identified gender (and marital status, living arrangements, nationality, education and employment status; data not reported). The terms ‘male’ and ‘female’ used in this article refer to self‐identified sex. DSM‐V MINI interviews for diagnosis of MDD, mania and psychosis, as well as demographic questionnaires, were administered as part of baseline screening and follow‐up. COVID‐19 lockdown status was assessed at follow‐up, based on the Australian Government's official lockdown dates and timestamps.

#### 2.4.2 Baseline Depressive Symptoms

The Quick Inventory of Depressive Symptomatology (QIDS SR‐16) (Rush et al. 2003) was used to assess depression at baseline and follow‐up (follow‐up data not reported). Questions in the QIDS correlate with mental disorder diagnostic symptoms, including sleep disturbance, sad mood, change in weight, concentration, self‐criticism, suicidal ideation, interest, energy and psychomotor agitation (Rush et al. 2003). Based on the sum of these domains, a total depressive score is calculated ranging from 0 to 27. The severity of depression can be based on this total score, with 1–5 = no depression, 6–10 = mild depression, 11–15 = moderate depression, 16–20 = severe depression, 21–27 = very severe depression (Rush et al. 2003).

#### 2.4.3 Daily Physical Activity

Participants were provided with an actigraphy device (GENEActiv watch, Activinsights Ltd., Kimbolton, United Kingdom), which provided continuous physical activity data. Participants were instructed to wear the actigraphy device on their wrist continuously and to remove it only when swapping it for the new one. A second actigraphy device was delivered to the participant after 3 weeks to replace the original device and prevent the battery from running out. Raw actigraphy data were processed with the open‐source R package GGIR (version 2.9–0) (Migueles et al. 2019). Minute‐to‐minute daily actigraphy data were derived per participant by summing five seconds of data (using Euclidean Norm Minus One [ENMO] metric). Minutes spent in light physical activity (physical activity level based on ENMO metric threshold of 30), moderate physical activity (physical activity level based on ENMO metric threshold of 100), vigorous physical activity (physical activity level based on ENMO metric threshold of 400) and sedentary time (time not spent in light, moderate or vigorous physical activity or sleep) were quantified from 12 am to 12 am for each day and each participant (Sabia et al. 2014; van Hees et al. 2013). Participants did not receive any feedback on their physical activity across the study period.

For analysis, we generated typical and daily physical activity levels. These were defined as:

*Daily activity*: Daily fluctuations in physical activity levels were person‐mean centred. This means that the daily fluctuation in physical activity was defined as the difference between the level of daily activity and each individual participant's mean value across the assessment period. Daily fluctuations were calculated for sedentary time, and light, moderate and vigorous physical activity (Hoffman 2015).

*Typical activity*: The average of daily light physical activity, moderate physical activity, vigorous physical activity and sedentary time over the full data collection period were entered as between‐subject variables, providing ‘typical’ (or average across the data collection period) levels. These were grand sample centred, reflecting the mean value for each person relative to the overall sample mean (Hoffman 2015).

### 2.5 Outcomes

#### 2.5.1 Daily Psychological Symptoms

The AWARE‐Light app was used to collect daily psychological symptoms, with participant responses being sent to a secure study database (Berkel et al. 2023). A randomly generated 128‐bit unique user ID was created for each participant after installation. AWARE‐Light prompted two EMA surveys each day (12 pm and 8 pm). Participants were given 2 h to respond to the survey, after which the survey was no longer available.

The evening survey examined daily positive affect (cheerful, happy, excited, relaxed), negative affect (sad, guilty, angry, nervous), stress and anxiety, scored on a scale of one to seven, with one being ‘not at all’ and seven being ‘very/extremely’, providing one stress score and one anxiety score per day for each participant (Watson, Clark, and Tellegen 1988). Positive and negative affect was assessed at 12 pm using the wording ‘from the time you woke up until now’, and 8 pm using the wording ‘from midday until now’. For our study, we averaged positive and negative affect across the two timepoints for a measure of daily affect. Stress and anxiety were only asked in the 8 pm survey using the wording ‘about your overall experiences today’. Positive and negative affect scores were calculated as the sum of all positive/negative mood constructs (four constructs, each scored from one to seven), providing a total positive affect score (/28) and a total negative affect score (/28) per day for each participant. Stress and anxiety were scored on a scale of one to seven.

### 2.6 Data Analysis

All analyses to identify associations between variables, confounders and outcomes were conducted using IBM SPSS Statistics for Windows, version 29.0 (IBM Corp., Armonk, NY, USA). A significance level of *α* = 0.05 was chosen to limit the probability of Type 1 error at 5%. Intra‐class coefficients (ICC) are reported for daily dependent variables.

Firstly, Pearson correlation coefficients were analysed to assess associations between age, sex, baseline depression (QIDS score), being in COVID‐19 lockdown (yes/no), light physical activity, moderate physical activity, vigorous physical activity and sedentary time, with EMA‐derived stress, anxiety, positive affect and negative affect. These were conducted to assess for multicollinearity between variables, covariates and outcomes for regression models.

Then, multi‐level linear mixed models with random intercepts for participants were used to examine within‐ and between‐person associations between physical activity and symptoms of stress, anxiety, positive affect and negative affect. A maximum likelihood estimation approach was used. In the first two models, light physical activity, moderate physical activity and vigorous physical activity, and sedentary time were added as variables for the outcomes of stress (model one) and anxiety (model two). In the third and fourth models, symptoms of positive and negative affect were the outcomes. Each physical activity/inactivity predictor was first included separately in models (e.g. light, moderate, vigorous, sedentary time in separate models). Then, to adjust for covariance between different activity types, we aimed to include all predictors in the model. However, due to high levels of multicollinearity between light and moderate physical activity, we ran the model twice (1) keeping light and removing moderate physical activity, and (2) removing light and keeping moderate physical activity.

In all models, within‐person variables (level one; light physical activity, moderate physical activity, vigorous physical activity and sedentary time) were person‐mean centred, providing ‘daily’ fluctuations. At level two, the average of daily light physical activity, moderate physical activity, vigorous physical activity, and sedentary time over the full data collection period were entered as between‐subject variables, providing ‘typical’ (or average across the data collection period) levels. Age, sex and baseline depression were also entered at level two as covariates in all models. For this analyses, sex refers to self‐identified sex, coding ‘males = 0’ and ‘females = 1’. For all models, the intercepts were random, and the slopes were fixed.

We also conducted a sensitivity analysis to explore models with COVID‐19 lockdown status as a covariate.

## 3 Results

### 3.1 Participants

**FIGURE 2:**
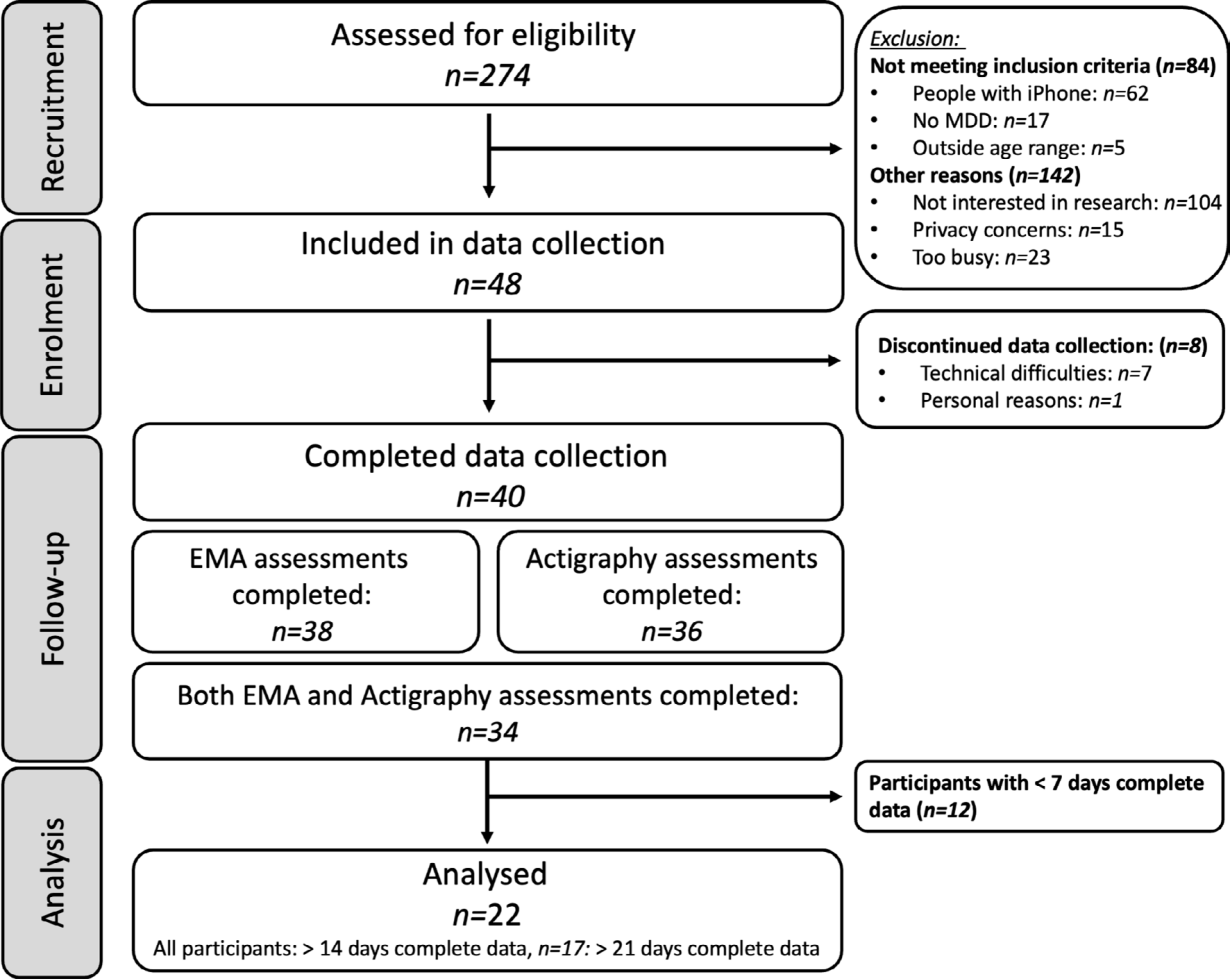
Participant flow diagram.

The reasons for the exclusion of participants at each phase of the study are reported in Figure 2. Overall, 274 participants were screened for eligibility. From these, 40 participants completed data collection, and 22 participants were included in the analysis, as they had more than 14 days of complete data (defined as days with both completed actigraphy and EMA data). From this selected sample, 77% had at least 21 days of complete data, with a total of 598 complete days of data collection across all participants. Further details of data completeness are reported in Table S2. Details of self‐reported medication usage, other medical conditions and current anxiety symptoms are reported in Table S3.

Participant characteristics, mean physical activity and psychological symptom ratings, and ICCs for daily data are reported in Table 1. Individual depressive symptom scores are reported in Table S4. Overall, 81.8% of participants had at least a moderate depressive score at baseline, with 54.6% in the severe and very severe depression category. On average over the eight‐week period, participants spent most of their time in sedentary (69.1%), and spent 19.1% in light physical activity, 11.4% in moderate physical activity and 0.5% in vigorous physical activity. We observed no significant differences in key study characteristics between those included and excluded from analyses (Table S5).

**TABLE 1:** Participant characteristics, baseline depression, mean physical activity and psychological ratings.

Participant characteristics	Mean ± SD/*N* (%)	Range (min‐max)	ICC
Age (years)	20.07 ± 3.12	16–25	—
Sex			
Female	17 (77.27%)	*—*	—
Male	5 (22.73%)	*—*	—
Gender			
Women	13 (59.09%)	*—*	—
Men	4 (18.18%)	*—*	—
Gender nonconforming	3 (13.64%)	*—*	—
Transgender	2 (9.09%)	*—*	—
COVID‐19 lockdown status			
Data collected during a lockdown	11 (50.00%)	*—*	—
Data collected during no lockdown	11 (50.00%)	*—*	—
Baseline depression score, QIDS (/27)	15.32 ± 5.58	3–26	—
Follow‐up depression score, QIDS (/27)	14.91 ± 5.49	3–27	—
EMA survey data			
Mean stress (/7)	3.62 ± 1.71	0–7	0.390
Mean anxiety (/7)	3.40 ± 1.67	0–7	0.407
Mean positive affect (/28)	12.50 ± 5.54	0–28	0.565
Mean negative affect (/28)	11.27 ± 4.94	3–26	0.453
Actigraphy data			
Mean light physical activity (min)	138.62 ± 63.59	12–410	0.618
Mean moderate physical activity (min)	81.24 ± 59.48	8–354	0.604
Mean vigorous physical activity (min)	3.22 ± 5.21	0–47	0.315
Mean sedentary time (min)	675.05 ± 133.24	318–1119	0.322

*Note:* scored /27, defined as 1–5 = no depression, 6–10 = mild depression, 11–15 = moderate depression, 16–20 = severe depression, 21–27 = very severe depression (Rush et al. 2003).

Abbreviation: QIDS: Quick Inventory of Depressive Symptomatology.

### 3.2 Correlations Between Demographics, Physical Activity and Psychological Outcomes

Table 2 presents correlations between the covariates and psychological outcomes. Age was significantly negatively correlated with average stress (*r* = −0.182; *p* < 0.001), anxiety (*r* = −0.190; *p* < 0.001), positive affect (*r* = −0.099; *p* = 0.015) and negative affect (*r* = −0.161; *p* < 0.001), with a younger age correlating with greater symptoms. Sex correlated with all psychological symptoms, with females reporting increased stress (*r* = 0.129; *p* = 0.002), anxiety (*r* = 0.145; *p* < 0.001) and negative affect (*r* = 0.111; *p* = 0.006) and decreased positive affect (*r* = −0.263; *p* < 0.001) compared with males. Baseline severity of depression scores (QIDS) was positively associated with stress (*r* = 0.198; *p* < 0.001), anxiety (*r* = 0.262; *p* < 0.001) and negative affect (*r* = 0.193; *p* < 0.001), and negatively associated with positive affect (*r* = −0.455, *p* < 0.001). COVID‐19 lockdown status significantly correlated with stress (*r* = −0.094; *p* = 0.021) and positive affect (*r* = −0.158; *p* < 0.001), with no lockdown increasing stress and positive affect.

**TABLE 2:** Pearson correlation coefficients between covariates, physical activity and psychological outcomes.

	Stress	Anxiety	Positive affect	Negative affect
Covariates				
Age	−0.182***	−0.190***	−0.099*	−0.161**
Sex	0.129**	0.145***	−0.263***	0.111**
Depression (baseline)	0.198***	0.262***	−0.455***	0.193***
COVID‐19 lockdown	−0.094*	0.012	−0.158***	−0.012
Physical activity				
Light physical activity (min)	−0.086*	−0.107**	0.210***	−0.108**
Moderate physical activity (min)	−0.077	−0.005	0.120**	−0.087*
Vigorous physical activity (min)	−0.153***	−0.100*	0.151***	−0.172***
Sedentary time (min)	0.062	0.033	−0.026	0.061

*Note:* ***p < 0.001, **p < 0.01, *p < 0.05.

Correlations between physical activity and psychological outcomes are shown in Table 2. Light physical activity negatively correlated with stress (*r* = −0.086, *p* = 0.036), anxiety (*r* = −0.107; *p* = 0.009) and negative affect (*r* = −0.108; *p* = 0.008), and positively correlated with positive affect (*r* = 0.210; *p* < 0.001). Moderate physical activity positively correlated with positive affect (*r* = 0.120; *p* = 0.003), and negatively correlated with negative affect (*r* = −0.087; *p* = 0.034) but no other psychological outcomes. Vigorous physical activity negatively correlated with stress (*r* = −0.153; *p* < 0.001), anxiety (*r* = −0.100; *p* = 0.014) and negative affect (*r* = −0.172; *p* < 0.001), and positively correlated with positive affect (*r* = 0.151; *p* < 0.001). Sedentary time did not significantly correlate with any psychological outcomes.

Correlations between physical activity intensities are reported in Table S6. Light and moderate physical activity were highly correlated. Therefore, only one of light or moderate physical activity is included in each model. No other correlations reached multicollinearity thresholds.

### 3.3 Associations Between Physical Activity and Psychological Symptoms

Table 3 presents the beta coefficients (*β*) and standard errors (SE) for typical physical activity (average across assessment period, level 2: between‐subject) and daily physical activity (daily fluctuations, level 1: within‐subject) on stress and anxiety. Table 4 presents the beta coefficients (*β*) and SE for typical physical activity (average across assessment period, level 2: between‐subject) and daily physical activity (daily fluctuations, level 1: within‐subject) on positive and negative affect. The beta coefficients (*β*) and SE for physical activity predicting stress, anxiety, positive affect and negative affect, with each intensity as a separate predictor are presented in Tables S7 and S8.

**TABLE 3:** Separate linear mixed models of physical activity (A) light, vigorous, sedentary, and (B) moderate, vigorous, sedentary on outcomes of stress (model 1) and anxiety (model 2).

	Model 1: stress	*p*	Model 2: anxiety	*p*
	*β* (SE)		*β* (SE)	
(A) LIGHT, vigorous, sedentary				
Intercept	**3.712 (0.593)**	**< 0.001**	**3.311 (0.609)**	**< 0.001**
Covariates				
Age	−0.104 (0.070)	0.154	−0.105 (0.072)	0.159
Sex	−0.166 (0.698)	0.814	0.087 (0.717)	0.905
Depression (baseline)	0.062 (0.055)	0.271	0.077 (0.057)	0.187
Level 1				
Daily light physical activity	**0.004 (0.001)**	**0.010**	**0.003 (0.001)**	**0.018**
Daily vigorous physical activity	−0.008 (0.013)	0.561	−0.006 (0.013)	0.637
Daily sedentary time	0.000 (0.001)	0.899	0.000 (0.000)	0.766
Level 2				
Typical light physical activity	0.001 (0.005)	0.830	0.001 (0.006)	0.808
Typical vigorous physical activity	**−0.152 (0.070)**	**0.041**	−0.096 (0.072)	0.196
Typical sedentary time	0.002 (0.003)	0.529	0.002 (0.003)	0.614
(B) MODERATE, vigorous, sedentary				
Intercept	**3.637 (0.612)**	**< 0.001**	**3.093 (0.608)**	**< 0.001**
Covariates				
Age	−0.107 (0.069)	0.138	−0.118 (0.069)	0.101
Sex	−0.083 (0.718)	0.909	0.328 (0.712)	0.649
Depression (baseline)	0.060 (0.048)	0.231	0.079 (0.048)	0.115
Level 1				
Daily moderate physical activity	**0.004 (0.002)**	**0.009**	**0.004 (0.001)**	**0.011**
Daily vigorous physical activity	−0.010 (0.013)	0.447	−0.008 (0.013)	0.506
Daily sedentary time	0.000 (0.001)	0.707	0.000 (0.001)	0.998
Level 2				
Typical moderate physical activity	0.003 (0.006)	0.616	0.008 (0.006)	0.178
Typical vigorous physical activity	**−0.158 (0.070)**	**0.035**	−0.109 (0.070)	0.133
Typical sedentary time	0.003 (0.003)	0.428	0.004 (0.003)	0.216

*Note:* *β*: unstandardised beta coefficient, SE: standard error. Level 1: within‐subject daily fluctuations, Level 2: between‐subject average across the assessment period. Bold indicates significant results.

**TABLE 4:** Separate linear mixed models of physical activity (A) light, vigorous, sedentary, and (B) moderate, vigorous, sedentary on outcomes of positive (model 3) and negative (model 4) affect.

	Model 3: positive affect	*p*	Model 4: negative affect	*p*
	*β* (SE)		*β* (SE)	
(A) LIGHT, vigorous, sedentary				
Intercept	**9.151 (1.896)**	**< 0.001**	**11.542 (2.076)**	**< 0.001**
Covariates				
Age	−0.329 (0.226)	0.159	−0.236 (0.247)	0.351
Sex	3.549 (2.233)	0.126	−0.865 (2.445)	0.727
Depression (baseline)	**−0.700 (0.176)**	**< 0.001**	0.206 (0.193)	0.297
Level 1				
Daily light physical activity	0.001 (0.004)	0.844	0.006 (0.004)	0.079
Daily vigorous physical activity	0.053 (0.032)	0.092	−0.062 (0.031)	0.051
Daily sedentary time	−0.001 (0.001)	0.530	0.001 (0.001)	0.480
Level 2				
Typical light physical activity	0.003 (0.018)	0.864	0.003 (0.019)	0.895
Typical vigorous physical activity	**0.526 (0.224)**	**0.028**	−0.388 (0.246)	0.129
Typical sedentary time	0.003 (0.009)	0.718	0.004 (0.010)	0.687
(B) MODERATE, vigorous, sedentary				
Intercept	**9.319 (1.969)**	**< 0.001**	**11.137 (2.136)**	**< 0.001**
Covariates				
Age	−0.310 (0.223)	0.179	−0.258 (0.242)	0.299
Sex	3.365 (2.309)	0.159	−0.418 (2.504)	0.869
Depression (baseline)	**−0.720 (0.155)**	**< 0.001**	0.209 (0.169)	0.229
Level 1				
Daily moderate physical activity	0.003 (0.004)	0.378	0.004 (0.004)	0.334
Daily vigorous physical activity	0.049 (0.032)	0.124	−0.061 (0.032)	0.055
Daily sedentary time	0.000 (0.001)	0.751	0.001 (0.001)	0.582
Level 2				
Typical moderate physical activity	−0.005 (0.018)	0.786	0.014 (0.020)	0.481
Typical vigorous physical activity	**0.533 (0.226)**	**0.028**	−0.410 (0.245)	0.108
Typical sedentary time	0.001 (0.010)	0.953	0.009 (0.011)	0.443

*Note:* *β*: unstandardised beta coefficient, SE: standard error. Level 1: within‐subject daily fluctuations, Level 2: between‐subject average across the assessment period. Bold indicates significant results.

#### 3.3.1 Stress

Typical vigorous physical activity was negatively associated with daily stress in both the light (*β*[95% CI] = −0.152 [−0.298, −0.007], *p* = 0.041) and moderate (*β*[95% CI] = −0.158 [−0.303, −0.012], *p* = 0.035) models (Table 3). In other words, higher levels of typical (average across the assessment period) vigorous physical activity were associated with lower levels of daily stress. No other typical physical activity levels, or covariates, were significantly associated with daily stress. Daily light physical activity (*β*[95% CI] = 0.004 [0.001, 0.006], *p* = 0.010) and daily moderate physical activity (*β*[95% CI] = 0.004 [0.001, 0.007], *p* = 0.009) were positively associated with daily stress, whereby higher levels of daily fluctuations in light and moderate physical activity were associated with higher levels of stress.

#### 3.3.2 Anxiety

Daily light physical activity (*β*[95% CI] = 0.003 [0.001, 0.006], *p* = 0.018) and daily moderate physical activity (*β*[95% CI] = 0.004 [0.001, 0.007], *p* = 0.011) were positively associated with daily anxiety, whereby higher levels of daily fluctuations light and moderate physical activity were associated with higher levels of anxiety (Table 3). No typical physical activities (average across the assessment period) were significantly associated with symptoms of anxiety.

#### 3.3.3 Positive Affect

Typical vigorous physical activity was associated with positive affect in both the light (*β*[95% CI] = 0.526 [0.061, 0.992], *p* = 0.028) and moderate (*β*[95% CI] = 0.533 [0.064, 1.002], *p* = 0.028) models (Table 4), meaning higher levels of typical (average across the assessment period) vigorous physical activity were associated with higher levels of daily positive affect. No other typical physical activity levels were associated with positive affect. No daily physical activity intensities were significantly associated with positive affect.

#### 3.3.4 Negative Affect

No typical or daily physical activity level (average across the assessment period), or covariates, were significantly associated with negative affect (Table 4).

### 3.4 Sensitivity Analysis: Inclusion of COVID‐19 Lockdown Status as a Covariate

The results of all sensitivity analyses with COVID‐19 lockdown as a covariate are reported in Tables S9–S10. The differences between the primary and sensitivity analyses are summarised in Table S11. Typical (average across assessment period) vigorous physical activity was no longer significantly associated with stress when considering COVID‐19 lockdown as a covariate.

## 4 Discussion

We examined associations between objectively assessed typical and daily light, moderate and vigorous physical activity, and sedentary time, and stress, anxiety, positive and negative affect in a young sample with depression. Results indicated that typical vigorous activity was associated with lower symptoms of stress and higher levels of positive affect. Daily light and moderate physical activity were positively associated with stress and anxiety. Sedentary time was not associated with symptoms of stress, anxiety, positive affect or negative affect. Given the available sample size, these associations should be validated in larger follow‐up studies. These results indicate differing associations between physical activity and psychological symptoms depending on the intensity (light, moderate and vigorous) and pattern (typical versus daily) of physical activity.

Only typical vigorous physical activity (average across the data collection period) was associated with symptoms of stress. This is not the first study to show that only higher intensity physical activity (i.e. vigorous) is associated with enhanced mental health (Nakagawa et al. 2020). The mechanisms by which physical activity benefits psychological symptoms include improved hypothalamic–pituitary–adrenal axis regulation, noradrenergic and serotoninergic effect, neurotrophic factor production and improved vascular function and oxygenation (Meeusen and De Meirleir 1995; Szuhany, Bugatti, and Otto 2015; Taylor, Aizenstein, and Alexopoulos 2013), and these neurobiological changes are more extensive and longer lasting at higher intensity activity (Nakagawa et al. 2020). However, the mean amount of vigorous physical activity detected in this study is low, and hence, future research should explore strategies to increase vigorous activity to confirm this relationship.

Higher levels of daily light and moderate physical activity were associated with higher levels of stress and anxiety. A possible explanation is that young people with MDD may increase their light and moderate physical activity as a coping mechanism when experiencing stress and anxiety, given that most intentional physical activity has been characterised as aerobic (light and moderate) intensity (Lin and Gao 2023). Motivation for physical activity seems to moderate the relationship between stress and physical activity, with prior research demonstrating a positive association in people with higher motivation for exercise (Nägel, Sonnentag, and Kühnel 2015). Therefore, given the similarity in model coefficients, the selection of light or moderate physical activity as a coping mechanism should consider person preference and stage of exercise engagement, to increase overall motivation and adherence. However, given the sample size and number of analyses, this may be a spurious finding, and further time‐lagged regression models should explore causality in this relationship. Therefore, future studies should continue to evaluate the relationship between daily and typical physical activity and symptoms of anxiety in young people with MDD.

Engaging in typical vigorous physical activity was significantly associated with improved symptoms of positive affect. This is in line with previous research showing physical activity was associated with less negative affect and more positive affect in healthy (Schultchen et al. 2019) and depressed populations (Mata et al. 2012) over 7 days. However, this was not replicated in inactive university students (Von Haaren et al. 2013) nor in a group of 10 depressed participants (Stavrakakis et al. 2015), yet individual variability and sample size had a substantial effect on significance (Stavrakakis et al. 2015). Only vigorous intensity was significantly associated with positive affect, again potentially due to greater stimulation of neurophysiological mechanisms which lead to improved mood (Nakagawa et al. 2020). Furthermore, a dose‐response of intensity has been evidenced only in depressed participants, whereby a higher intensity increased affect more than lower intensity activity (Mata et al. 2012), which may be driving this significant association. Therefore, strategies to increase typical levels of vigorous physical activity could be useful to increase positive affect in young people with MDD.

In our study, being sedentary was not significantly associated with any psychological outcomes. There is a lack of evidence investigating associations between sedentary time and psychological outcomes (Wright, Williams, and Veldhuijzen van Zanten 2023). However, it is important to consider the different types of sedentary behaviour. For example, sedentary time which involves being mentally active (e.g. office‐based work) has a different impact on psychological health compared with mentally passive sedentary time (e.g. watching television) (Hallgren et al. 2020). Furthermore, negative health behaviours accompanying sedentary time such as poor diet and smoking also play a role in psychological well‐being and are often more prevalent in people experiencing psychological distress (George et al. 2022). Therefore, future study should investigate the role of different types of sedentary time on psychological outcomes, especially in populations with depression.

The present study conducted a sensitivity analysis with COVID‐19 lockdown status as a covariate in analyses. Typical vigorous physical activity was no longer significantly associated with lower symptoms of stress. This may be driven by the level of vigorous physical activity undertaken by participants who were enrolled during a lockdown, as this was potentially higher than in those who were not experiencing a lockdown. This is contradictory to a review presenting reduced physical activity across all intensities during the pandemic (Wilke et al. 2022). However, this review notes that Australia was the only country not to show a pandemic‐induced reduction in physical activity (Wilke et al. 2022). Furthermore, there is evidence suggesting that individuals undertaking less physical activity before the pandemic increased their physical activity engagement during the pandemic, whilst individuals who were physically active before the pandemic struggled to maintain their activity habits (Elliott et al. 2022). As physical activity engagement is generally lower in depressed populations (Minaeva et al. 2020), perhaps our sample was able to maintain or improve their physical activity behaviours during the lockdown. However, future research is required to understand the influence of a pandemic on the relationship between physical activity and psychological symptoms, to better protect our physical and psychological health during periods of social isolation.

There are several strengths in this study. First, physical activity was assessed objectively. Most previous studies have used self‐reported physical activity, which is less precise in determining the intensity, amount and duration of physical activity than objective assessments (Gianfredi et al. 2020). Furthermore, we were able to obtain real‐time reports of psychological symptoms, using EMA. The use of EMA allows investigation of within and between‐subject associations, and we applied rigorous statistical methods, implementing regression models that also account for the interrelationships of observations within participants (Shiffman, Stone, and Hufford 2008). Finally, the inclusion of a young clinical population with a confirmed diagnosis of MDD provides a novel exploration into physical activity behaviour and psychological outcomes. Although mood and physical activity has been investigated previously using EMA in adults with depression, the data collection period was limited to 5 days with only one single‐item assessment of mood (Hollands et al. 2020). By assessing objective physical activity and psychological outcomes (including stress, anxiety and affect) for up to 8 weeks, we can develop a more realistic picture of physical activity behaviour in this population.

This study has some limitations. First, our sample was slightly older (i.e. mean age of 21 years compared to 18 years within headspace centres) and had a higher proportion of females (i.e. 77% compared to 62% within headspace centres), which may limit the generalisability of the findings (Rickwood et al. 2023). However, this is common with EMA research (Wright, Williams, and Veldhuijzen van Zanten 2023), which may limit generalisability of the findings to males, especially as physical activity behaviours, and its effect on depression, are influenced by sex (Zhang and Yen 2015). We did not track the treatments each participant received. However, in the centres from which participants were recruited, psychotherapy is considered first‐line care for the management of depression, with cognitive behavioural therapy most commonly delivered at our recruitment centres (Hetrick et al. 2015). Therefore, treatment across the study period was likely relatively homogenous. Although 40 participants were recruited, only 22 participants were included in our study. Hence, we may be underpowered to detect between‐person associations, and this should be considered during the interpretation of results. The reason for our sample size was to allow for a greater number of observations with both actigraphy and EMA data completed across the day, which was not possible by including all participants. We opted to not complete multiple‐testing correction of *p*‐values due to the exploratory nature of our study (Lakens 2022; Rothman 1990), therefore, these associations should be interpreted with caution and future research should use EMA to investigate these findings in confirmatory research with larger samples. Furthermore, it is important to acknowledge the role of the COVID‐19 pandemic in our findings, with 50% of participants experiencing a governmental lockdown during data collection. However, including lockdown as a covariate in sensitivity analyses is an interesting exploration, and future research should further investigate how physical activity and psychological outcomes are affected by a pandemic. Finally, although we consider a range of covariates in our statistical models, other measures such as body mass index, not collected in our study, could be important to consider.

## 5 Conclusion

The present study showed that typical vigorous physical activity is associated with lower symptoms of stress and higher levels of positive affect in young people with MDD. Daily light and moderate physical activity were associated with higher levels of daily stress and anxiety. Although these findings are exploratory, they present a complex and nuanced relationship between physical activity and psychological symptoms. Further research is required to clearly define the causal and daily relationships between physical activity and stress, anxiety and positive and negative affect.

## Disclosure

The funders had no role in the study design, data collection, data analysis, data interpretation or writing of this manuscript.

## Ethics Statement

University of Melbourne Human Research Ethics (ID: 1955691.4).

## Conflicts of Interest

The authors declare no conflicts of interest.

## Supporting information


Data S1.

## Data Availability

Not all participants in this study provided consent for their data to be openly shared. For the remaining participants, data may be made available upon reasonable request to the corresponding author.
