# Supplementary material for: The Dynamic Association Between Physical Activity and Psychological Symptoms in Young People With Major Depressive Disorder: An Active and Passive Sensing Longitudinal Cohort Study
Source: Early Interv Psychiatry. 2025 Feb 23;19(3):e70018. doi: 10.1111/eip.70018 (PMC11847758; doi:10.1111/eip.70018)
Supplement: Supplementary file 1 — Data S1. [file EIP-19-0-s001.docx]

**Supplementary material**

**Supplementary Table 1.** STROBE Statement—checklist of items that should be included in reports of observational studies

|  | | | **Item No.** | **Recommendation** | | | | **Page No.** | | | | **Relevant section** | |  |
| --- | --- | --- | --- | --- | --- | --- | --- | --- | --- | --- | --- | --- | --- | --- |
| **Title and abstract** | | | 1 | (*a*) Indicate the study’s design with a commonly used term in the title or the abstract | | | | 1 | | | | Title | |  |
|  |  |  |  | (*b*) Provide in the abstract an informative and balanced summary of what was done and what was found | | | | 3 | | | | Abstract | |  |
| **Introduction** | | | | | | | | | | | |  | |  |
| Background/rationale | | | 2 | Explain the scientific background and rationale for the investigation being reported | | | | 4-5 | | | | Introduction | |  |
| Objectives | | | 3 | State specific objectives, including any prespecified hypotheses | | | | 5 | | | | Introduction (paragraph 4) | |  |
| **Methods** | | | | | | | | | | | |  | |  |
| Study design | | | 4 | Present key elements of study design early in the paper | | | | 6 | | | | Methods (paragraph 1) | |  |
| Setting | | | 5 | Describe the setting, locations, and relevant dates, including periods of recruitment, exposure, follow-up, and data collection | | | | 6-7 | | | | Methods (Procedures) | |  |
| Participants | | | 6 | (*a*) *Cohort study*—Give the eligibility criteria, and the sources and methods of selection of participants. Describe methods of follow-up  *Case-control study*—Give the eligibility criteria, and the sources and methods of case ascertainment and control selection. Give the rationale for the choice of cases and controls  *Cross-sectional study*—Give the eligibility criteria, and the sources and methods of selection of participants | | | | 6 | | | | Methods (Participants) | |  |
|  |  |  |  | (*b*) *Cohort study*—For matched studies, give matching criteria and number of exposed and unexposed  *Case-control study*—For matched studies, give matching criteria and the number of controls per case | | | |  | |  | | | |  |
| Variables | | | 7 | Clearly define all outcomes, exposures, predictors, potential confounders, and effect modifiers. Give diagnostic criteria, if applicable | | | | 7-9 | | | | Methods (Variables and confounders; Outcomes) | |  |
| Data sources/ measurement | | | 8* | For each variable of interest, give sources of data and details of methods of assessment (measurement). Describe comparability of assessment methods if there is more than one group | | | | 6-9 | | | | Methods (Participants; Variables and confounders; Outcomes) | |  |
| Bias | | | 9 | Describe any efforts to address potential sources of bias | | | | 6 | | | | Methods (Participants) | |  |
| Study size | | | 10 | Explain how the study size was arrived at | | | | 24 | | | | Figure 1 | |  |
| Quantitative variables | | 11 | | | | Explain how quantitative variables were handled in the analyses. If applicable, describe which groupings were chosen and why | 7-9 | | | | | | Methods (Variables and confounders; Outcomes) | |
| Statistical methods | | 12 | | | | (*a*) Describe all statistical methods, including those used to control for confounding | 10 | | | | | | Methods (Data analysis) | |
|  |  |  |  |  |  | (*b*) Describe any methods used to examine subgroups and interactions |  | | | | | | Not applicable | |
|  |  |  |  |  |  | (*c*) Explain how missing data were addressed | 6,10 | | | | | | Methods (Participants; Data analysis) | |
|  |  |  |  |  |  | (*d*) *Cohort study*—If applicable, explain how loss to follow-up was addressed  *Case-control study*—If applicable, explain how matching of cases and controls was addressed  *Cross-sectional study*—If applicable, describe analytical methods taking account of sampling strategy |  | | | | | | Not applicable | |
|  |  |  |  |  |  | (*e*) Describe any sensitivity analyses | 10 | | | | | | Methods (Data analysis) | |
| **Results** | | | | | | | | | | | | | | |
| Participants | | 13* | | | | (a) Report numbers of individuals at each stage of study—eg numbers potentially eligible, examined for eligibility, confirmed eligible, included in the study, completing follow-up, and analysed | 24 | | | | | | Results (Participants); Figure 1 | |
|  |  |  |  |  |  | (b) Give reasons for non-participation at each stage | 24 | | | | | | Results (Participants); Figure 1 | |
|  |  |  |  |  |  | (c) Consider use of a flow diagram | 24 | | | | | | Figure 1 | |
| Descriptive data | | 14* | | | | (a) Give characteristics of study participants (eg demographic, clinical, social) and information on exposures and potential confounders | 26 | | | | | | Results (Participants); Table 1 | |
|  |  |  |  |  |  | (b) Indicate number of participants with missing data for each variable of interest |  | | | | | | Not applicable | |
|  |  |  |  |  |  | (c) *Cohort study*—Summarise follow-up time (eg, average and total amount) | 11 | | | | | | Results (Participants) | |
| Outcome data | | 15* | | | | *Cohort study*—Report numbers of outcome events or summary measures over time | 26 | | | | | | Table 1 | |
|  |  |  |  |  |  | *Case-control study—*Report numbers in each exposure category, or summary measures of exposure |  | | | | | | Not applicable | |
|  |  |  |  |  |  | *Cross-sectional study—*Report numbers of outcome events or summary measures |  | | | | | | Not applicable | |
| Main results | | 16 | | | | (*a*) Give unadjusted estimates and, if applicable, confounder-adjusted estimates and their precision (eg, 95% confidence interval). Make clear which confounders were adjusted for and why they were included | 11-12 28-29 | | | | | | Results (Associations between physical activity and psychological symptoms) | |
|  |  |  |  |  |  | (*b*) Report category boundaries when continuous variables were categorized |  | | | | | | Not applicable | |
|  | |  | | | | (*c*) If relevant, consider translating estimates of relative risk into absolute risk for a meaningful time period |  | | | | | | Not applicable | |
| Other analyses | 17 | | | | Report other analyses done—eg analyses of subgroups and interactions, and sensitivity analyses | | | | 14 | | Results (Sensitivity analysis) | | | |
| **Discussion** | | | | | | | | | | | | | | |
| Key results | 18 | | | | Summarise key results with reference to study objectives | | | | 15 | | Discussion (paragraph 1) | | | |
| Limitations | 19 | | | | Discuss limitations of the study, taking into account sources of potential bias or imprecision. Discuss both direction and magnitude of any potential bias | | | | 15  18-19 | | Discussion (paragraph 1, limitations, conclusions) | | | |
| Interpretation | 20 | | | | Give a cautious overall interpretation of results considering objectives, limitations, multiplicity of analyses, results from similar studies, and other relevant evidence | | | | 18 | | Discussion (limitations) | | | |
| Generalisability | 21 | | | | Discuss the generalisability (external validity) of the study results | | | | 18 | | Discussion (limitations) | | | |
| **Other information** | | | | |  | | | | | | | | | |
| Funding | 22 | | | | Give the source of funding and the role of the funders for the present study and, if applicable, for the original study on which the present article is based | | | | 2 | | Funding | | | |

| **Supplementary Table 2.** Number of days of actigraphy and EMA data collected for each participant | | | |
| --- | --- | --- | --- |
| **Participant** | **Days of actigraphy** | **Days of EMA** | **Days of both actigraphy + EMA** |
| 001 | 52 | 54 | 50 |
| 002 | 53 | 18 | 16 |
| 003 | 49 | 36 | 28 |
| 004 | 43 | 41 | 36 |
| 005 | 21 | 52 | 21 |
| 006 | 41 | 41 | 31 |
| 007 | 44 | 20 | 17 |
| 008 | 20 | 47 | 17 |
| 009 | 29 | 42 | 26 |
| 010 | 39 | 35 | 28 |
| 011 | 43 | 23 | 22 |
| 012 | 38 | 44 | 26 |
| 013 | 41 | 38 | 31 |
| 014 | 43 | 33 | 30 |
| 015 | 37 | 40 | 29 |
| 016 | 50 | 31 | 30 |
| 017 | 36 | 56 | 31 |
| 018 | 51 | 31 | 27 |
| 019 | 43 | 30 | 25 |
| 020 | 49 | 57 | 48 |
| 021 | 35 | 26 | 15 |
| 022 | 21 | 38 | 14 |
| Note: Participant in this table does not reflect the random participant code assigned for the study. | | | |

| **Supplementary Table 3.** Self-reported medication type, dose, and usage, concurrent medical conditions, and anxiety symptoms of included participants. | | | | | |
| --- | --- | --- | --- | --- | --- |
| **Participant** | **Medication type** | **Medication dose + frequency** | **Other medical conditions** | **DASS Anxiety Subscale Score** | **DASS Anxiety Subscale Summary** |
| 001 | Fluoxetine | 20mg/daily | - | 26 | Extremely Severe |
| 002 | - | - | - | 12 | Moderate |
| 003 | Escitalopram | 20mg/daily | - | 14 | Moderate |
| 004 | Methylphenidate  Venlafaxine | 27mg/daily  150mg/daily | ADHD | 18 | Severe |
| 005 | - | - | - | 20 | Extremely Severe |
| 006 | Venlafaxine  Intuniv | 225ml/daily  2ml/daily | ADHD | 10 | Moderate |
| 007 | Escitalopram  Pantoprazole | 10ml/daily  20ml/daily | - | 24 | Extremely Severe |
| 008 | Fluoxetine | 40ml/daily | - | 14 | Moderate |
| 009 | Fluoxetine | 60ml/daily | - | 22 | Extremely Severe |
| 010 | Zaleplon  Melatonin  Pantoprazole | 7.5ml/daily  10ml/daily  20ml/As needed | Back pain  Sleep disorder (unclear) | 14 | Moderate |
| 011 | Desvenlafaxine  Valproate  Melatonin | 150mg/daily  400mg/daily  10mg/daily | Insomnia | 16 | Severe |
| 012 | Metformin  Sertraline | 500mg/twice a day  200mg/daily | Anxiety  Stigmatism | 36 | Extremely Severe |
| 013 | Fluoxetine  Catapres | 40mg/daily  Not reported | PTSD  Anxiety  ADHD  Sleep disorder (unclear) | 22 | Extremely Severe |
| 014 | - | - | - | 18 | Severe |
| 015 | Fluoxetine  Melatonin  Meloxicam | 20mg/daily  2mg/daily  7.5mg/as needed | Anxiety  Fibromyalgia  Sleep disorder (unclear) | 30 | Extremely Severe |
| 016 | - | - | - | 34 | Extremely Severe |
| 017 | - | - | - | 26 | Extremely Severe |
| 018 | - | - | - | 22 | Extremely Severe |
| 019 | Fluoxetine | 20ml/daily | - | 12 | Moderate |
| 020 | Sertraline | 150ml/daily | - | 6 | Normal |
| 021 | - | - | - | 14 | Moderate |
| 022 | - | - | - | 10 | Moderate |
| Note: Participant in this table does not reflect the random participant code assigned for the study and medication type, dose and frequency, and co-morbid disorders were self-reported by participants.  - are NA/not reported.  NSAID = non-steroidal anti-inflammatory, DASS = Depression Anxiety and Stress Scale.  Cut-offs for DASS anxiety are based on Levibond & Levibond 1995. | | | | | |

| **Supplementary Table 4.** Baseline and follow up depression score (QIDS). | | |
| --- | --- | --- |
| **Participant** | **Baseline depression score** | **Follow up depression score** |
| 001 | 20 | 16 |
| 002 | 11 | 11 |
| 003 | 18 | 18 |
| 004 | 21 | 18 |
| 005 | 13 | 10 |
| 006 | 21 | 18 |
| 007 | 14 | 9 |
| 008 | 26 | 27 |
| 009 | 16 | 13 |
| 010 | 18 | 19 |
| 011 | 14 | 16 |
| 012 | 20 | 24 |
| 013 | 20 | 12 |
| 014 | 13 | 17 |
| 015 | 17 | 14 |
| 016 | 20 | 21 |
| 017 | 13 | 15 |
| 018 | 16 | 14 |
| 019 | 8 | 14 |
| 020 | 9 | 13 |
| 021 | 6 | 6 |
| 022 | 3 | 3 |
| QIDS: Quick Inventory of Depressive Symptomatology; scored /27. The severity of depression can be based on this total score, with 1-5 = no depression, 6-10 = mild depression, 11-15 = moderate depression, 16-20 = severe depression, 21-27 = very severe depression (Rush et al., 2003). Note: Participant in this table does not reflect the random participant code assigned for the study. | | |

| **Supplementary Table 5.** Participant characteristics and differences between included and excluded participants. | | | |
| --- | --- | --- | --- |
| **Participant Characteristics** | **Included (n=22)** | **Excluded (n=18)** | **p** |
|  | Mean ± SD / N (*%*) | Mean ± SD / N (*%*) |  |
| **Age (years)** | 20.23 ± 3.25 | 21.06 ± 2.39 | 0.374 |
| **Sex** |  |  | 0.279 |
| Female | *17* | *11* |  |
| Male | *5* | *7* |  |
| **Gender** |  |  | 0.298 |
| Women | 13 *(59.09 %)* | *9 (50.00 %)* |  |
| Men | 4 *(18.18 %)* | *6 (33.33 %)* |  |
| Gender nonconforming | 3 *(13.64 %)* | *1 (5.55 %)* |  |
| Transgender | 2 *(9.09 %)* | *2 (11.11 %)* |  |
| **COVID-19 lockdown status** |  |  | 0.495 |
| Data collected during a lockdown | 11 *(50.00 %)* | *11 (61.11 %)* |  |
| Data collected during no lockdown | 11 *(50.00 %)* | *7 (38.89 %)* |  |
| **Baseline depression score, QIDS (/27)** | 15.32 ± 5.58 | 16.06 ± 4.30 | 0.649 |
| **Follow up depression score, QIDS (/27)** | 14.91 ± 5.49 | 15.11 ± 5.66 | 0.910 |
| QIDS: Quick Inventory of Depressive Symptomatology; scored /27, defined as 1-5 = no depression, 6-10 = mild depression, 11-15 = moderate depression, 16-20 = severe depression, 21-27 = very severe depression (Rush et al., 2003). | | | |

| **Supplementary Table 6**. Pearson correlation coefficients between physical activity intensities | | | | |
| --- | --- | --- | --- | --- |
|  | Daily Light Physical Activity | Daily Moderate Physical Activity | Daily Vigorous Physical Activity | Daily Sedentary Time |
| Daily Light Physical Activity | N/A | 0.808*** | 0.109** | –0.329*** |
| Daily Moderate Physical Activity | 0.808*** | N/A | 0.252*** | –0.441*** |
| Daily Vigorous Physical Activity | 0.109** | 0.252*** | N/A | –0.087* |
| Daily Sedentary Time | –0.329*** | –0.441*** | –0.087* | N/A |
|  | Daily Light Physical Activity (person centred) | Daily Moderate Physical Activity (person centred) | Daily Vigorous Physical Activity (person centred) | Daily Sedentary Time  (person centred) |
| Daily Light Physical Activity (person centred) | N/A | 0.779*** | 0.107** | –0.292*** |
| Daily Moderate Physical Activity (person centred) | 0.779*** | N/A | 0.169*** | –0.375*** |
| Daily Vigorous Physical Activity (person centred) | 0.107** | 0.169*** | N/A | –0.029 |
| Daily Sedentary Time (person centred) | –0.292*** | –0.375*** | 0.029 | N/A |
|  | Daily Light Physical Activity (grand centred) | Daily Moderate Physical Activity (grand centred) | Daily Vigorous Physical Activity (grand centred) | Daily Sedentary Time  (grand centred) |
| Daily Light Physical Activity (grand centred) | N/A | 0.829*** | 0.120** | –0.406*** |
| Daily Moderate Physical Activity (grand centred) | 0.829*** | N/A | 0.370*** | –0.563*** |
| Daily Vigorous Physical Activity (grand centred) | 0.120** | 0.370*** | N/A | –0.210*** |
| Daily Sedentary Time (grand centred) | –0.406*** | –0.563*** | –0.210*** | N/A |

Person centred: defined as the difference between the level of daily activity and the participants mean value

Grand centred: defined as the mean value for each person relative to the overall sample mean

*** P<0.001, ** P<0.01, *P<0.05

| **Supplementary Table 7.** Separate linear mixed models of physical activity on outcomes of stress (model 1) and anxiety (model 2), with each activity intensity entered separately. Intercepts and covariates are not reported here given they are different across the separate models. | | | | |
| --- | --- | --- | --- | --- |
|  | **Model 1: Stress**  **B (SE)** | **p** | **Model 2: Anxiety**  **B (SE)** | **p** |
| ***Level 1*** |  |  |  |  |
| Daily Light Physical Activity | **0.003 (0.001)** | **0.009** | **0.003 (0.001)** | **0.012** |
| Daily Moderate Physical Activity | **0.004 (0.001)** | **0.010** | **0.004 (0.001)** | **0.008** |
| Daily Vigorous Physical Activity | –0.004 (0.013) | 0.770 | –0.002 (0.013) | 0.847 |
| Daily Sedentary Time | 0.000 (0.000) | 0.524 | 0.000 (0.000) | 0.309 |
| ***Level 2*** |  |  |  |  |
| Typical Light Physical Activity | –0.002 (0.005) | 0.740 | –0.001 (0.005) | 0.867 |
| Typical Moderate Physical Activity | **–**0.002 (0.005) | 0.627 | 0.002 (0.004) | 0.732 |
| Typical Vigorous Physical Activity | **–0.161 (0.069)** | **0.030** | –0.103 (0.071) | 0.162 |
| Typical Sedentary Time | 0.002 (0.003) | 0.342 | 0.002 (0.002) | 0.492 |
| B: Unstandardised beta coefficient, SE: standard error.  Level 1: within-subject daily fluctuations, level 2: between-subject average across assessment period. | | | | |

| **Supplementary Table 8.** Separate linear mixed models of physical activity on outcomes of positive affect (model 3) and negative affect (model 4), with each activity intensity entered separately. Intercepts and covariates are not reported here given they are different across the separate models. | | | | |
| --- | --- | --- | --- | --- |
|  | **Model 3: Positive affect**  **B (SE)** | **p** | **Model 4: Negative affect**  **B (SE)** | **p** |
| ***Level 1*** |  |  |  |  |
| Daily Light Physical Activity | 0.002 (0.003) | 0.599 | 0.005 (0.003) | 0.155 |
| Daily Moderate Physical Activity | 0.005 (0.004) | 0.175 | 0.002 (0.004) | 0.611 |
| Daily Vigorous Physical Activity | 0.054 (0.031) | 0.084 | –0.056 (0.031) | 0.075 |
| Daily Sedentary Time | –0.001 (0.001) | 0.456 | 0.000 (0.001) | 0.803 |
| ***Level 2*** |  |  |  |  |
| Typical Light Physical Activity | 0.002 (0.016) | 0.893 | –0.004 (0.017) | 0.809 |
| Typical Moderate Physical Activity | 0.001 (0.015) | 0.934 | –0.002 (0.016) | 0.917 |
| **Typical Vigorous Physical Activity** | **0.511 (0.221)** | **0.030** | –0.408 (0.243) | 0.107 |
| Typical Sedentary Time | –0.001 (0.008) | 0.911 | 0.006 (0.009) | 0.504 |
| B: Unstandardised beta coefficient, SE: standard error.  Level 1: within-subject daily fluctuations, level 2: between-subject average across assessment period. | | | | |

| **Supplementary Table 9.** Multilevel modelling coefficients of physical activity on stress and anxiety: adjusted for COVID‑19 lockdown as a covariate | | | |
| --- | --- | --- | --- |
|  | **Model 1: Stress (B(SE), p)** | | **Model 2: Anxiety (B(SE), p)** |
| ***A) LIGHT, vigorous, sedentary*** |  | |  |
| Intercept | **3.735 (.581), p <.001** | | **3.320(.608), p <.001** |
| ***Covariates*** |  | |  |
| Age | –.107(.069), p =.136 | | –.106(.072), p =.154 |
| Sex | .113(.739), p =.880 | | .196(.773), p =.802 |
| Depression (baseline) | .075(.056), p =.191 | | –.082(.058), p =.171 |
| COVID lockdown | –.497 (.506), p =.337 | | –.196(.530), p =.715 |
| ***Level 1*** |  | |  |
| Daily Light Physical Activity | **.004(.001), p =.010** | | **.003(.001), p =.018** |
| Daily Vigorous Physical Activity | –.008(.013), p =.560 | | –.006(.013), p =.637 |
| Daily Sedentary Time | .000(.001), p =.899 | | .000(.000), p =.766 |
| ***Level 2*** |  | |  |
| Typical Light Physical Activity | –.002(.005), p =.760 | | .002(.006), p =.782 |
| Typical Vigorous Physical Activity | –.103(.085), p =.239 | | –.077(.089), p =.400 |
| Typical Sedentary Time | .002(.003), p =.433 | | .002(.003), p =.578 |
| ***B) MODERATE, vigorous, sedentary*** | |  |  |
| Intercept | **3.633(.597), p <.001** | | **3.090(.602), p <.001** |
| ***Covariates*** |  | |  |
| Age | –.111(.068), p =.116 | | –.120(.068), p =.092 |
| Sex | .260(.768), p =.738 | | .533(.744), p =.499 |
| Depression (baseline) | .072(.049), p =.151 | | .086(.049), p =.092 |
| COVID lockdown | –.551(.509), p =.290 | | –.329(.513), p =.529 |
| ***Level 1*** |  | |  |
| Daily Moderate Physical Activity | **.004(.002), p =.009** | | .**004(.001), p =.011** |
| Daily Vigorous Physical Activity | –.010(.013), p =.446 | | –.008(.013), p =.506 |
| Daily Sedentary Time | .000(.001), p =.707 | | .000(.001), p =.998 |
| ***Level 2*** |  | |  |
| Typical Moderate Physical Activity | .004(.006), p =.481 | | .009(.006), p =.148 |
| Typical Vigorous Physical Activity | –.105(.084), p =.227 | | –.077(.085), p =.372 |
| Typical Sedentary Time | .003(.003), p =.307 | | .004(.003), p =.177 |
| B: Unstandardised beta coefficient, SE: standard error.  Level 1: within-subject daily fluctuations, level 2: between-subject average across assessment period. | | | |

| **Supplementary Table 10.** Multilevel modelling coefficients of physical activity on positive and negative affect: adjusted for COVID-19 lockdown as a covariate | | | |
| --- | --- | --- | --- |
|  | **Model 3: Positive affect (B(SE), p)** | | **Model 4: Negative affect  (B(SE), p)** |
| ***A) LIGHT, vigorous, sedentary*** |  | |  |
| Intercept | **9.288(1.796), p <.001** | | **11.554(2.077), p <.001** |
| ***Covariates*** |  | |  |
| Age | –.344(.214), p =.121 | | –.237(.248), p =.349 |
| Sex | **4.987(2.286), p =.040** | | –.747(2.644), p =.780 |
| Depression (baseline) | **–.633(.172), p =.001** | | .212(.199), p =.298 |
| COVID lockdown | –2.595(1.570), p =.113 | | –.212(1.822), p =.908 |
| ***Level 1*** |  | |  |
| Daily Light Physical Activity | .001(.004), p =.878 | | .006(.004), p =.079 |
| Daily Vigorous Physical Activity | .053(.031), p =.092 | | –.062(.031), p =.051 |
| Daily Sedentary Time | –.001(.001), p =.530 | | .001(.001), p =.480 |
| ***Level 2*** |  | |  |
| Typical Light Physical Activity | .006(.017), p =.743 | | .003(.019), p =.887 |
| Typical Vigorous Physical Activity | **.785(.264), p =.007** | | –.367(.305), p =.243 |
| Typical Sedentary Time | .006(.009) p =.530 | | .004(.010), p =.678 |
| ***B) MODERATE, vigorous, sedentary*** | |  |  |
| Intercept | **9.310(1.869), p <.001** | | **11.135(2.133), p <.001** |
| ***Covariates*** |  | |  |
| Age | –.329(.212), p =.135 | | –.261(.242), p =.293 |
| Sex | 4.944(2.403), p =.051 | | –.148(2.743), p =.958 |
| Depression (baseline) | **–.660(.152), p <.001** | | .219(.174), p =.221 |
| COVID lockdown | –2.562(1.598), p =.123 | | –.439(1.828), p =.812 |
| ***Level 1*** |  | |  |
| Daily Moderate Physical Activity | .003(.004), p =.375 | | .004(.004), p =.333 |
| Daily Vigorous Physical Activity | .049(.032), p =.124 | | –.061(.032), p =.055 |
| Daily Sedentary Time | .000(.001), p =.751 | | .001(.001), p =.582 |
| ***Level 2*** |  | |  |
| Typical Moderate Physical Activity | .000(.018), p =.985 | | .015(.020), p =.461 |
| Typical Vigorous Physical Activity | **.779(.264), p =.007** | | –.368(.301), p =.235 |
| Typical Sedentary Time | .004(.010) p =.683 | | .009(.011), p =.423 |
| B: Unstandardised beta coefficient, SE: standard error.  Level 1: within-subject daily fluctuations, level 2: between-subject average across assessment period. | | | |

| **Supplementary Table 11.** Multilevel modelling coefficients: summary of changes of physical activity predictors across models adjusted for COVID-19 lockdown as a covariate. | | | | | | | |
| --- | --- | --- | --- | --- | --- | --- | --- |
|  | **Model 1: Stress** | | **Model 2: Anxiety** | | **Model 3: Positive affect** | | **Model 4: Negative affect** |
| ***Influenced Parameters:***  🡪 adjusted B(SE), p | | | | | | | |
| Typical Vigorous Physical Activity ***(Level 2)*** | | ***In light model:***  –.152 (0.070), p =.041*🡪  –.103(.085), p =.239**^N.S^**  ***In moderate model:***  –.158 (0.070), p =.035 🡪  –.105(.084), p =.227 **^N.S^** | | Nil | | Nil | Nil |
| SE: standard error, N.S: not significant. ***p<.001, **p<.01, *p<.05. Level 1: within-subject daily fluctuations, level 2: between-subject average across assessment period. | | | | | | | |
